# HIV-1 prevalence and factors associated with infection in the conflict-affected region of North Uganda

**DOI:** 10.1186/1752-1505-1-3

**Published:** 2007-03-01

**Authors:** Massimo Fabiani, Barbara Nattabi, Chiara Pierotti, Filippo Ciantia, Alex A Opio, Joshua Musinguzi, Emintone O Ayella, Silvia Declich

**Affiliations:** 1 National Centre for Epidemiology, Surveillance and Health Promotion, Istituto Superiore di Sanità, Rome, Italy; 2 St. Mary's Hospital Lacor, Gulu, Uganda; 3 AVSI, Kampala, Uganda; 4 National Diseases Control Department, Ministry of Health, Kampala, Uganda

## Abstract

**Background:**

Since 1986, northern Uganda has been severely affected by civil strife with most of its population currently living internally displaced in protected camps. This study aims at estimating the HIV-1 prevalence among this population and the factors associated with infection.

**Methods:**

In June-December 2005, a total of 3051 antenatal clinics attendees in Gulu, Kitgum and Pader districts were anonymously tested for HIV-1 infection as part of routine sentinel surveillance. Factors associated with the infection were evaluated using logistic regression models.

**Results:**

The age-standardised HIV-1 prevalence was 10.3%, 9.1% and 4.3% in the Gulu, Kitgum and Pader district, respectively. The overall prevalence in the area comprised of these districts was 8.2% when data was weighted according to the districts' population size. Data from all sites combined show that, besides older women [20–24 years: adjusted odds ratio (AOR) = 1.96, 95% confidence interval (CI): 1.29–2.97; 25–29 years: AOR = 2.01, 95% CI: 1.30–3.11; ≥ 30 years: AOR = 1.91, 95% CI: 1.23–2.97], unmarried women (AOR = 1.47, 95% CI: 1.06–2.04), and those with a partner with a non-traditional occupation (AOR = 1.62, 95% CI: 1.18–2.21), women living outside of protected camps for internally displaced persons have a higher risk of being HIV-1 infected than internally displaced women (AOR = 1.55, 95% CI: 1.15–2.08).

**Conclusion:**

Although published data from Gulu district show a declining HIV-1 prevalence trend that is consistent with that observed at the national level since 1993, the prevalence in North Uganda is still high. Internally displaced women have a lower risk of being infected probably because of their reduced mobility and accessibility, and increased access to health prevention services.

## Introduction

In sub-Saharan Africa, the HIV epidemic is commonly monitored through the sentinel surveillance of pregnant women attending antenatal clinics (ANC), which provides important indications for planning and evaluating public-health interventions.

In Uganda, a national HIV-1 sentinel surveillance system has existed for more than 10 years and currently involves 20 ANCs [1]. However, few of these ANCs are located in the north, where the available data on the HIV-1 epidemic are still limited and where the violent civil strife that has affected this area for almost two decades has had a great impact on the health profile of the population [2,3].

The ANC of the St. Mary's Hospital Lacor is located in the Gulu district of North Uganda and has participated in the national HIV-1 sentinel surveillance system since 1993. In 2005, the ANCs of the St. Joseph's Hospital (Kitgum district) and Dr Ambrosoli Memorial Hospital (Pader district), both located in northern Uganda, also participated in the national sentinel surveillance system. The population living in the area comprised of the districts of Gulu, Kitgum and Pader (referred to as "Acholi region") accounts for almost half of the population living in the North-Central region of Uganda, which also includes the districts of Lira and Apac [4]. About 10% of people living in the Acholi region are resident in urban areas and approximately 90% are internally displaced in protected camps as a consequence of the civil conflict that affects northern Uganda since 1986. In February 2005, there was an estimated population of over 1 000 000 internally displaced persons (IDP) in the Acholi region, who were forced into the currently existing 96 protected camps mainly as a consequence of the increased rebel activities in 1996–1997 and 2002–2004 [5]. Most of the IDP have a reduced mobility and access to lands for cultivating, thus basing their subsistence on food aid from international organisations.

We analysed the HIV-1 surveillance data from ANCs in the Acholi region with the objective of increasing the available information on the HIV-1 epidemic in northern Uganda and identifying the socio-demographic factors associated with HIV-1 infection in this conflict-affected region.

## Materials and methods

The unlinked and anonymous HIV-1 surveillance at the ANCs of St. Mary's Hospital Lacor, St. Joseph's Hospital and Dr. Ambrosoli Memorial Hospital was implemented by the "Istituto Superiore di Sanità" (the National Institute of Health of Italy) and AVSI, in collaboration and with the approval of the STD/AIDS Control Programme of the Ugandan Ministry of Health and the hospitals' ethical committees.

All first-time attendees of the ANCs of the St. Mary's Hospital Lacor, St. Joseph's Hospital and Dr Ambrosoli Memorial Hospital are routinely offered voluntary counselling and testing for HIV-1 infection and asked for verbal consent to interview as part of the national programme for the prevention of mother-to-child transmission of HIV-1 infection. In the period June-December 2005, a total of 3976 women out of 4135 women who consecutively attended the clinics (96.2%) were interviewed. Information on their socio-demographic characteristics was collected through a questionnaire administered by specifically trained midwives. All but 17 first-time attendees were tested for syphilis infection as part of the routine antenatal care provided at these sites. For an age-stratified random sample of 1190 out of the 1970 consecutive ANC attendees of St. Mary's Hospital Lacor (June-November 2005), for all the 833 consecutive ANC attendees of the St. Joseph's Hospital (June-December 2005), and for all the 1156 consecutive ANC attendees of Dr. Ambrosoli Memorial Hospital (June-September 2005), leftover sera from the syphilis test were anonymously tested for HIV-1 after having removed any possible identifier. Unlinked and anonymous testing of ANC attendees is routinely used for HIV surveillance purposes in most African countries with generalised epidemics. The woman's consent to HIV testing is not required where blood is taken for other purposes (e.g., syphilis test) and leftover sera are stripped of all identifying markers [6]. This minimises the bias introduced when women refuse to allow their blood to be tested for HIV infection. At the St. Mary's Hospital Lacor, as recommended in the guidelines for second generation HIV surveillance developed by the Joint United Nations Programme on HIV/AIDS (UNAIDS) and the World Health Organization (WHO), the sera tested for HIV-1 infection were over-sampled from women aged 15–24 years, among whom changes in prevalence more closely reflect changes in incidence [6]. The serum samples were tested at on-site laboratories using an algorithm based on rapid tests: samples were first tested with Capillus (Trinity Biotech plc, Bray, Co., Wicklow, Ireland); reactive sera were then re-tested for confirmation with Serocard (Trinity Biotech plc, Bray, Co. Wicklow, Ireland.); and discordant samples were tested with Multispot (Bio-Rad, Marnes La Coquette, France).

The statistical analyses were conducted excluding data for the 128 women with missing information on age or displacement status, thus limiting the analyses to 3051 records. For each site, the HIV-1 prevalence was calculated by directly standardizing by age, using as reference the distribution of women in the Ugandan female population of reproductive age derived from the 2002 census data. The overall prevalence for the Acholi region was calculated by weighting the site-specific data according to the districts' population size. Data from all sites combined were analysed to evaluate the association between HIV-1 infection and the socio-demographic factors considered in this study (i.e., age, displacement status, education, occupation, marital status, age and occupation of partner, and parity). A univariate analysis was performed using the Pearson's chi-square test or the Yates' corrected chi-square test, when appropriate. The factors associated with HIV-1 infection were then evaluated in multivariate analysis using logistic regression models. The adjusted odds ratios (AOR) and their 95% confidence intervals (CI) were used to describe the strength of the associations. In order to avoid the over-adjustment for variables that are likely to mediate the effect of certain factors on HIV-1 infection (e.g., occupation is likely to mediate the effect of education on HIV-1 infection), we considered five hierarchical levels in designing multivariate analysis [7]: 1) age group and displacement status were included in the first model; 2) education was added in the second model; 3) occupation was added in the third model; 4) marital status and age and occupation of partner were added in the fourth model; and, finally, 5) parity was added in the last model. At each level, only variables associated with HIV-1 infection at a P-level less than 0.20 were retained in the following models as potential confounders. All models were run by also controlling for site of testing. In order to evaluate possible differences in the risk profile of pregnant women who are internally displaced in protected camps compared with pregnant women living outside of protected camps, the interaction terms between displacement status and each of the other factors included in the multivariate models were tested trough the log-likelihood ratio test.

## Results

Pregnant women anonymously tested for HIV-1 infection at the three ANC sites did not greatly differ according to the socio-demographic factors presented in Table 1. The only differences were observed in Pader district, where almost all pregnant women were internally displaced in protected camps and most of them had a partner that was an agricultural worker, and in the Gulu district, where we observed a lower proportion of married women partly because of the sampling design adopted in this site (i.e., over-sampling of women aged 15–19 years).

**Table 1:** Socio-demographic characteristics of the antenatal clinic attendees anonymously tested for HIV-1 infection in Gulu, Kitgum and Pader districts (North Uganda)

	Gulu (n = 1190)	Kitgum (n = 730)	Pader (n = 1131)	Overall (n = 3051)
Mean Age (SD)	24.1 (6.1)	25.2 (5.7)	25.3 (6.0)	24.8 (6.0)
Internally displaced (%)	558 (46.9)	385 (52.7)	1080 (95.5)	2023 (66.3)
Primary or lower education (%)	944 (79.4)	571 (79.7)	1019 (91.6)	2534 (84.0)
Traditional occupation^a ^(%)	1067 (89.9)	658 (91.8)	1077 (97.8)	2802 (93.2)
Married (%)	417 (35.2)	539 (76.3)	973 (87.5)	1929 (64.3)
Mean age of partner (SD)	29.5 (7.6)	30.8 (7.1)	30.2 (7.4)	30.0 (7.5)
Partner with traditional occupation^a ^(%)	544 (46.0)	299 (47.8)	802 (73.8)	1645 (56.8)
Primipara (%)	363 (30.5)	172 (23.9)	243 (21.6)	778 (25.7)

^a ^Traditional occupation: agricultural worker and housewife.

The age-standardised HIV-1 prevalence was higher in the Gulu district (10.3%) and Kitgum district (9.1%) compared with Pader district (4.3%). When data was weighted according to the districts' population size, the overall prevalence in the Acholi region was estimated at 8.2%, with the highest prevalence among women in the 20–29 years age group (Table 2). Overall, women who were internally displaced in protected camps had a reduced HIV-1 prevalence compared with women living outside of protected camps (6.3% vs 11.6%). This difference was observed for each age group and testing site, with the only exception of women aged less than 20 years or 25–29 years in Pader district, where the age-specific prevalence estimates for women living outside of protected camps were based on a very small sample size.

**Table 2:** HIV-1 prevalence by age group, site and displacement status among the 3051 antenatal clinic attendees in Gulu, Kitgum and Pader districts (North Uganda)

	HIV-1 prevalence (number of women tested)				
	
	< 20 years	20–24 years	25–29 years	≥ 30 years	Overall^a^
Gulu district					
IDP	4.9 (164)	10.3 (145)	10.3 (116)	8.3 (133)	8.2 (558)
Not IDP	6.6 (183)	15.4 (201)	13.5 (133)	13.9 (115)	12.3 (632)
Total	5.8 (347)	13.3 (346)	12.0 (249)	10.9 (248)	10.3 (1190)
Kitgum district					
IDP	6.9 (72)	6.3 (112)	10.5 (95)	8.5 (106)	8.0 (385)
Not IDP	7.8 (51)	8.3 (133)	14.8 (81)	11.3 (80)	10.3 (345)
Total	7.3 (123)	7.3 (245)	12.5 (176)	9.7 (186)	9.1 (730)
Pader district					
IDP	2.7 (188)	4.8 (335)	3.8 (264)	4.8 (293)	4.1 (1080)
Not IDP	0.0 (11)	19.2 (26)	0.0 (11)	33.3 (3)	10.5 (51)
Total	2.5 (199)	5.8 (361)	3.6 (275)	5.1 (296)	4.3 (1131)
Overall^a^					
IDP	4.4 (424)	6.8 (592)	7.4 (475)	6.8 (532)	6.3 (2023)
Not IDP	6.6 (245)	13.2 (360)	13.5 (225)	13.2 (198)	11.6 (1028)
Total	5.2 (669)	9.5 (952)	9.6 (700)	8.8 (730)	8.2 (3051)

IDP, internally displaced women

^a ^HIV-1 prevalence calculated weighting data according to the population distribution by district and age derived from the 2002 Uganda Census.

The univariate analysis of data from all sites combined showed that education was the only variable for which a statistically significant association with HIV-1 infection was not found, although the prevalence was somewhat higher among more educated women (Table 3).

**Table 3:** Factors associated with HIV-1 infection among the 3051 antenatal clinic attendees in Gulu, Kitgum and Pader districts (North Uganda)

	Univariate analysis			Multivariate analysis	
		
	N	HIV-1 prevalence (95% CI)	P-value	Adjusted OR^a ^(95% CI)	P-value
Age group (1)			0.022		
< 20 years	669	5.1 (3.5–7.0)		1	
20–24 years	952	8.9 (7.2–10.9)		1.96 (1.29–2.97)	0.002
25–29 years	700	8.9 (6.9–11.2)		2.01 (1.30–3.11)	0.002
≥ 30 years	730	8.2 (6.3–10.5)		1.91 (1.23–2.97)	0.004
Internally displaced (1)			< 0.001		
Yes	2023	6.0 (5.0–7.2)		1	
No	1028	11.6 (9.7–13.7)		1.55 (1.15–2.08)	0.004
Education (2)			0.142		
Primary or lower	2534	7.6 (6.6–8.7)		1	
Secondary or higher	484	9.7 (7.2–12.7)		0.95 (0.66–1.37)	0.791
Occupation^b ^(3)			0.023		
Traditional	2802	7.6 (6.6–8.6)		1	
Modern	203	12.3 (8.1–17.6)		1.07 (0.67–1.71)	0.767
Marital status (4)			< 0.001		
Married	1929	6.6 (5.5–7.8)		1	
Not married	1072	10.4 (8.6–12.3)		1.47 (1.06–2.04)	0.021
Age of partner (4)			0.008		
< 25 years	690	5.1 (3.6–7.0)		1	
25–34 years	1479	8.5 (7.1–10.1)		1.35 (0.83–2.20)	0.226
≥ 35 years	826	9.0 (7.1–11.1)		1.56 (0.85–2.87)	0.151
Occupation of partner^b ^(4)			< 0.001		
Traditional	1645	5.6 (4.5–6.8)		1	
Modern	1251	10.8 (9.1–12.6)		1.62 (1.18–2.21)	0.003
Parity (5)			0.027		
Primipara	778	5.9 (4.4–7.8)		1	
Multipara	2254	8.5 (7.4–9.7)		1.29 (0.83–1.99)	0.255

OR, odds ratio; CI, confidence interval; numbers in brackets near the variable names indicate the hierarchical level assigned to each factor in multivariate analysis (from 1 to 5).

^a ^OR adjusted for site of testing and all factors assigned to the same hierarchical level and those associated with HIV-1 infection at a P-level < 0.20 in the previous levels;^b ^Traditional occupation: agricultural worker and housewife; modern occupation: clerk, business woman/man, professional, soldier, student and other.

In the multivariate analysis, associations were found for increased age (20–24 years: AOR = 1.96, 95% CI: 1.29–2.97; 25–29 years: AOR = 2.01, 95% CI: 1.30–3.11; ≥ 30 years: AOR= 1.91, 95% CI: 1.23–2.97), residence outside of protected camps for IDP (AOR = 1.55, 95% CI: 1.15–2.08), being unmarried (AOR = 1.47, 95% CI: 1.06–2.04), and modern occupation of partner (i.e., clerk, businessman, professional, soldier, student or other than agricultural worker) (AOR = 1.62, 95% CI: 1.18–2.21) (Table 3). When running the same logistic regression analyses separately for each ANC site, associations were found for all of the above variables for all ANC sites, although these associations were sometimes not statistically significant because of the reduced statistical power due to stratification (data not shown); no additional variables were found to be significantly associated with HIV-1 infection, although, in Pader district, the associations with high level of education (AOR = 1.85, 95% CI: 0.79–4.35) and modern occupation of the woman (AOR = 1.95, 95% CI: 0.51–7.47) appeared stronger than in the overall analysis.

According to the results of the multivariate analysis by displacement status (Table 4), among women who were living in protected camps for IDP, high level of education (AOR = 2.29, 95% CI: 1.30–4.04), modern occupation of the woman (AOR = 3.62, 95% CI: 1.32–9.91), and modern occupation of the partner (AOR = 2.38, 95% CI: 1.60–3.53) were significantly associated with HIV-1 infection. Among women who were living outside of protected camps, significant associations were found for increased age (20–24 years: AOR = 2.25, 95% CI: 1.24–4.09; 25–29 years: AOR = 2.29, 95% CI: 1.21–4.35; ≥ 30 years: AOR= 2.27, 95% CI: 1.18–4.39), low level of education (AOR = 0.64, 95% CI: 0.42–1.00), and being unmarried (AOR = 2.08, 95% CI: 1.31–2.30). When testing for interactions, significant differences in the HIV-1 risk profile between women who were living in protected camps and those who were living outside of protected camps were found in relation to education (likelihood ratio test, P = 0.001), occupation of woman (likelihood ratio test, P = 0.016), occupation of partner (likelihood ratio test, P = 0.003), and marital status (likelihood ratio test, P = 0.084), although the latter interaction was of borderline significance.

**Table 4:** Factors associated with HIV-1 infection among the antenatal clinic attendees living in/out of protected camps for internally displaced people in Gulu, Kitgum and Pader districts (North Uganda)

	Internally displaced (n = 2023)		No internally displaced (n = 1028)	
		
	Adjusted OR^a ^(95% CI)	P-value	Adjusted OR^a ^(95% CI)	P-value
Age group				
< 20 years	1		1	
20–24 years	1.71 (0.96–3.05)	0.070	2.25 (1.24–4.09)	0.008
25–29 years	1.79 (0.98–3.25)	0.057	2.29 (1.21–4.35)	0.011
≥ 30 years	1.68 (0.93–3.04)	0.085	2.27 (1.18–4.39)	0.015
Education*				
Primary or lower	1		1	
Secondary or higher	2.29 (1.30–4.04)	0.004	0.64 (0.42–1.00)	0.050
Occupation^b,^*				
Traditional	1		1	
Modern	3.62 (1.32–9.91)	0.012	0.86 (0.51–1.43)	0.554
Marital status*				
Married	1		1	
Not married	0.97 (0.60–1.57)	0.889	2.08 (1.31–2.30)	0.002
Occupation of partner^b,^*				
Traditional	1		1	
Modern	2.38 (1.60–3.53)	< 0.001	0.96 (0.61–1.52)	0.865

OR, odds ratio; CI, confidence interval.

^a ^OR adjusted as for the overall analysis (see Table 3). Only factors significantly associated with HIV-1 infection in at least one group are presented; ^b ^Traditional occupation: agricultural worker and housewife; modern occupation: clerk, business woman/man, professional, soldier, student and other;

* Factors with a significant risk difference between the IDP group and the no-IDP group according to the interaction test (log-likelihood ratio test).

## Discussion

Published data from the Gulu district show a declining HIV-1 prevalence trend that is consistent with that observed at the national level (from 26.0 in 2003 to 11.3 in 2003) [1,8,9]. However, despite this decline, the prevalence among pregnant women in the Acholi region of North Uganda is still high, especially considering that this is mainly a rural area with about 10% of its population living in urban settings. In fact, the HIV-1 prevalence in the Acholi region is higher than the rates reported at ANC sites in other rural areas of Uganda (median = 4.5% in 2002, range: 0.7%-7.6%) and it is also higher than the rates reported at ANC sites in urban areas (median = 7.2% in 2002, range: 5.0%-10.8%) [1]. In general, this high prevalence can probably be attributed to the effects of the civil strife that has affected the region since 1986, namely the social and economic crises, food shortages, and reduced access to health care and prevention services.

However, the prevalence of HIV-1 infection is not homogeneous across the three districts comprised in the Acholi region. In fact, Gulu district and Kitgum district showed a prevalence that is higher compared with that observed in the Pader district, partly because, according to the 2002 Uganda census, a higher percentage of the population in the former districts live in urban areas (25.1% and 14.8% in the Gulu district and Kitgum district, respectively, compared with 2.7% in the Pader district) [4], a condition often found to be associated with an increased risk of being HIV-1 infected [9-11]. Moreover, a higher percentage of pregnant women tested in the Pader district were internally displaced in protected camps (Table 1), a condition that, independently on age and district of residence, has been shown to be associated with a reduced risk of being HIV-1 infected (Tables 2, 3).

When interpreting the results of this study, it should be considered that estimates of HIV-1 prevalence based on data from ANCs likely represent an underestimate of the prevalence among the general female population [11-17]. This is mainly because HIV-positive women have a reduced fertility compared to HIV-negative women, as a result of biological and socio-behavioural factors, and are thus under-represented in ANCs [16-19]. However, the HIV prevalence derived from ANC data is usually assumed to closely approximate the prevalence in the overall general population (males and females combined) and is thus used as input to estimate national prevalence level and trends [14,20-22]. This assumption is supported by findings from the recent population-based HIV-1 serosurvey conducted in Uganda in 2004–2005, which showed a HIV-1 prevalence among men and women aged 15–49 years in the general population of North-Central Uganda that is equal to that observed among the ANC attendees in our study (8.2%) [23].

A potential bias in our study is that related to possible differences in ANC attendance between HIV-positive and HIV-negative women, which could make pregnant women attending ANCs not representative of pregnant women in the general population. However, this bias probably did not greatly affect the results of our study, given that in northern Uganda 92% of pregnant women have been reported to attend ANCs for a first visit, although this estimate could be biased because of the limited reliability of self-reported information and the possible scarce inclusion of IDPs in the survey from which it is derived [24].

About one-third of pregnant women included in the study lived outside of protected camps compared with approximately 10% of the whole Acholi population. This is because two out of three ANCs included in this study are located within municipalities and are thus likely to capture mostly women living in towns or in the closest surrounding camps. Given that residence outside of protected camps has been found to be associated with HIV-1 infection, this could have introduced a bias toward an over-estimation of the HIV-1 prevalence in the region's population. Moreover, given that access to these ANCs is reduced among IDPs, it is possible that a selection bias has been introduced because of the different access of IDPs with different risk of being HIV-1 infected. In general, this study is based on data from only one ANC in each of the three districts in the Acholi region. As a consequence, the results reflect the HIV prevalence in the hospitals' catchment areas and may be not fully representative of the whole region's population.

With regard to the factors associated with HIV-1 infection, the strength and direction of the associations found in the univariate analysis are consistent with findings from other studies conducted in sub-Saharan Africa, where significant associations have been found for socio-demographic factors such as increased age, modern occupation, and being unmarried [10,11,14,15]. However, when controlling for potential confounders in the multivariate analysis, age group, displacement status, marital status, and occupation of the partner were found to be the only factors significantly associated with HIV-1 infection.

While most of these associations were diffusely investigated in the past, few studies, in our knowledge, have attempted to measure the association between HIV and displacement in sub-Saharan Africa [25,26]. Our findings show that people who are internally displaced in protected camps have a risk of being HIV-infected that is reduced by one-third with respect to people living outside of protected camps. This is a quite unexpected results, given that the overcrowding, the poor hygienic, nutritional and socio-economic conditions, the increased risk of sexual violence and abuse, and the strict contact with the military are commonly thought to increase the risk of HIV-1 transmission among IDP [27-30]. However, recent analyses have highlighted how the relationship between HIV-1 infection and forced displacement is probably more complex, suggesting that the reduced mobility and accessibility, and the increased access to health, education and prevention services among IDP may balance or overcome the HIV-related risks mentioned above [31,32]. Moreover, the "protective" effect of displacement is expected to increase with its duration. In fact, although the initial phase of displacement is likely to determine a high-risk context for HIV-1 transmission, the prolonged time of isolation and the implementation of education and preventive services might reduce the risk of HIV-infection among people who are internally displaced in protected camps. At the same time, people continuing to live outside of protected camps have a higher mobility and are concentrated in urban settings, conditions that have been often found to be associated with a high risk of HIV-1 infection [9-11]. Information on the duration of displacement were not collected in this survey and therefore it has been not possible to assess the relationship between this factor and the risk of being HIV-1 infected.

Although the risk profile derived from multivariate analyses did not differ among the three districts, it differs between the group of women who were internally displaced and those who were not internally displaced. High level of education and non-traditional occupation of woman and partner appear to be risk factors only for internally displaced women, among whom these conditions are likely to be associated with a relative increased mobility and thus a potentially increased exposure to infection. By contrast, being unmarried was found to be associated with HIV-1 infection only among women who live outside of protected camps, probably because, independently on marital status, the risk-behaviours usually related to this condition (e.g., mobility) are reduced among women living in protected camps.

The conceptual framework utilised in the multivariate analysis (i.e., the hierarchical classification of variables into five different levels according to assumptions on their causal relationships) could be questionable in some cases [7]. In fact, for some factors, the causal pathway leading to their association with HIV-1 infection is not always clear (e.g., marital status could mediate the effect of occupation on HIV-1 infection and vice versa). However, no important differences in results were observed when multivariate models were run using different hierarchical classifications or simultaneously including all the factors in the multivariate model.

In conclusion, although the HIV-1 prevalence trend in the Gulu District is consistent with that observed at the national level, the HIV-1 prevalence in the Acholi region is still high. The most conspicuous factors found to be associated with HIV-1 infection in this study are age, marital status, occupation of partner, and displacement status. People who are internally displaced in protected camps showed a reduced risk of being HIV-1 infected compared with those who are not internally displaced, thus bringing into question the common assumption on a positive association between HIV-1 infection and displacement. Further studies are needed to adequately evaluate the complex relationship between HIV-1 infection and internal displacement, including serial HIV-1 prevalence surveys and behavioural surveillance among both displaced and non-displaced populations.
